# High Prevalence of Intra-Familial Co-colonization by Extended-Spectrum Cephalosporin Resistant *Enterobacteriaceae* in Preschool Children and Their Parents in Dutch Households

**DOI:** 10.3389/fmicb.2018.00293

**Published:** 2018-02-21

**Authors:** Apostolos Liakopoulos, Gerrita van den Bunt, Yvon Geurts, Martin C. J. Bootsma, Mark Toleman, Daniela Ceccarelli, Wilfrid van Pelt, Dik J. Mevius

**Affiliations:** ^1^Department of Bacteriology and Epidemiology, Wageningen Bioveterinary Research, Lelystad, Netherlands; ^2^Julius Center for Health Sciences and Primary Care, University Medical Center Utrecht, Utrecht, Netherlands; ^3^Centre for Infectious Disease Control, National Institute for Public Health and the Environment (RIVM), Bilthoven, Netherlands; ^4^Department of Mathematics, Faculty of Science, Utrecht University, Utrecht, Netherlands; ^5^Division of Infection and Immunity, School of Medicine, Cardiff University, Cardiff, United Kingdom; ^6^Department of Infectious Diseases and Immunology, Faculty of Veterinary Medicine, Utrecht University, Utrecht, Netherlands

**Keywords:** *Escherichia coli*, ESBL/AmpC, Netherlands, plasmid, insertion sequence, household, co-carriage

## Abstract

Extended-spectrum cephalosporin-resistant (ESC^R^) *Enterobacteriaceae* pose a serious infection control challenge for public health. The emergence of the ESC^R^ phenotype is mostly facilitated by plasmid-mediated horizontal extended-spectrum β-lactamases (ESBLs) and AmpC gene transfer within *Enterobacteriaceae*. Current data regarding the plasmid contribution to this emergence within the Dutch human population is limited. Hence, the aim of this study was to gain insight into the role of plasmids in the dissemination of ESBL/AmpC genes inside Dutch households with preschool children and precisely delineate co-colonization. In 87 ESC^R^ *Enterobacteriaceae* from fecal samples of parents and preschool children within 66 Dutch households, genomic localization, plasmid type and insertion sequences linked to ESBL/AmpC genes were determined. Chromosomal location of ESBL/AmpC genes was confirmed when needed. An epidemiologically relevant subset of the isolates based on household co-carriage was assessed by Multilocus Sequence Typing and Pulsed-Field Gel Electrophoresis for genetic relatedness. The narrow-host range I1α and F plasmids were the major facilitators of ESBL/AmpC-gene dissemination. Interestingly, we documented a relatively high occurrence of chromosomal integration of typically plasmid-encoded ESBL/AmpC-genes. A high diversity of non-epidemic *Escherichia coli* sequence types (STs) was revealed; the predominant STs belonged to the pandemic lineages of extraintestinal pathogenic *E. coli* ST131 and ST69. Intra-familiar co-carriage by identical ESC^R^ *Enterobacteriaceae* was documented in 7 households compared to 14 based on sole gene typing, as previously reported. Co-carriage was more frequent than expected based on pure chance, suggesting clonal transmission between children and parents within the household.

## Introduction

Extended-spectrum cephalosporin-resistant (ESC^R^) *Enterobacteriaceae* have emerged worldwide as a significant cause of hospital-, health care- and community-associated infections (Paterson and Bonomo, 2005; Jacoby, 2009; Pitout, 2013; Woerther et al., 2013). The increase in prevalence of ESC^R^ phenotype, which has been observed in the last decades, is mainly due to the production of extended-spectrum β-lactamases (ESBLs) and to a lesser degree to acquired AmpC β-lactamases (Paterson and Bonomo, 2005; Jacoby, 2009; Pitout, 2013; Woerther et al., 2013). ESBL and AmpC β-lactamases belong to different structural and functional classification groups (Ambler, 1980; Bush et al., 1995), resulting in differences in their hydrolytic spectrums. Yet, they are both able to hydrolyse the oxyimino-β-cephalosporins commonly used in clinical practice, such as cefotaxime and ceftazidime (Paterson and Bonomo, 2005; Jacoby, 2009). The emergence of the ESC^R^ phenotype is facilitated mostly by plasmid-mediated horizontal transfer of ESBL and AmpC genes within *Enterobacteriaceae* (Carattoli, 2009).

Transmission of ESC^R^ *Enterobacteriaceae* within households and subsequently amongst the community has been documented to occur in multiple ways: from patients with community-acquired infections (Valverde et al., 2008), patients recently discharged or cared for in a hospital (Lo et al., 2010; Mihaila et al., 2010; Hilty et al., 2012; Löhr et al., 2013; Haverkate et al., 2017), infants colonized after neonatal ICU admission (Strenger et al., 2013), adopted children from countries with high prevalence of ESC^R^ *Enterobacteriaceae* (Tande et al., 2010) and from international travelers to their household contacts (Arcilla et al., 2017).

Several studies in the Netherlands have reported the predominance of *bla*_CTX−M_ genes in ESC^R^ *Enterobacteriaceae*, mostly *Escherichia coli*, among hospital and primary care patients (van der Bij et al., 2011; Voets et al., 2012; Reuland et al., 2013), nursing home residents (Willemsen et al., 2015) and humans in the community (van Hoek et al., 2015; van den Bunt et al., 2016). Among them, *E. coli* belonging to sequence types (ST)10, ST38, ST69 and ST131 recovered from Dutch individuals have been recently associated with ESC^R^ phenotype (Leverstein-van Hall et al., 2011; Overdevest et al., 2011, 2015; van der Bij et al., 2011; Voets et al., 2012; Reuland et al., 2013, 2016; Huijbers et al., 2014; Dohmen et al., 2015; van Hoek et al., 2015; Willemsen et al., 2015; Souverein et al., 2016; Voor in 't holt et al., 2016). However, data regarding the genetic background, plasmid replicon types and Insertion Sequence (IS) of ESBL/AmpC genes and their plasmid-mediated dissemination among the Dutch human population have been scarcely investigated.

We previously reported the initial results of a 2-year cross-sectional study where ESC^R^ *Enterobacteriaceae* incidence in Dutch households with preschool children was investigated (van den Bunt et al., 2016). Co-occurrence of ESC^R^ phenotypes (14.6%) between children and their parents was solely defined based on the presence of the same ESBL/AmpC gene(s), mostly *bla*_CTX−M_ and *bla*_SHV−12_. In order to delineate co-colonization within the household in a more accurate way, we extended the analysis to molecular typing of the *Enterobacteriaceae* isolates and the genetic background of the ESBL/AmpC genes. Adding plasmid and strain typing revealed more stringent intra-familial co-colonization rates, compared to the gene-based typing, and these results are reported here.

## Materials and methods

### Bacterial isolates

Eighty-seven ESC^R^ *Enterobacteriaceae* (*E. coli, Klebsiella pneumoniae* and *Enterobacter cloacae*) isolated from fecal samples of parents and preschool children within 66 households (van den Bunt et al., 2016) were included in this study. Previously uncharacterized isolates of the same species recovered from the same fecal sample as distinctive colonial morphotypes were added to this study (isolates a and b, Tables 1, 2). Isolates recovered from child and parent were designated as C and P, respectively. Pure isolates were stored at −80°C in Peptone Broth supplemented with 30% (v/v) glycerol. This study is part of a project that received ethics approval from the Medical Research Ethics Committee of Utrecht University (WAG/om/13/048247). Informed consent was obtained from all subjects.

**Table 1:** Genetic characteristics of ESC^R^ *Enterobacteriaceae* recovered from parents or children in Dutch households.

**Household**	Isolate^*^	**Bacterial species**	**ESBL/AmpC genes^∧^**	**Location**	**Plasmid rep/inc-type**	**Plasmid subtype**	**Insertion sequence**
1	C-12371	*E. coli*	*bla*_TEM−52var_	Plasmid	X1	NA	IS*26*
2	C-13309	*E. coli*	*bla*_SHV−12_	Plasmid	I1α	pST3/pCC3	IS*26*
3	C-13311	*E. coli*	*bla*_CMY−2_	Plasmid	I1α	pST43	IS*Ecp1*
4	C-20046	*E. coli*	*bla*_CTX−M−15_	Plasmid	F	F2:A4:B1	IS*Ecp1*
5	C-20895	*E. coli*	*bla*_CMY−2_	Plasmid	I1γ	pST189	IS*Ecp1*
6	C-21281a	*E. coli*	*bla*_CTX−M−14_	Chromosome	–	–	IS*Ecp1*
7	C-24053	*E. cloacae*	*bla*_CTX−M−3_	Chromosome	–	–	IS*Ecp1*
8	C-24900	*E. coli*	*bla*_CTX−M−15_	Chromosome	–	–	IS*Ecp1*
9	C-25932	*E. coli*	*bla*_CTX−M−15_	Plasmid	NT		NP
10	C-26971	*E. coli*	*bla*_SHV−12_	Plasmid	I1α	pST95	IS*26*
11	C-29568	*E. coli*	*bla*_CTX−M−15_	Plasmid	HI2	pST3	IS*Ecp1*
12^§^	C-29929a	*E. coli*	*bla*_CTX−M−1_	Plasmid	I1α	pST35	IS*Ecp1*
	C-29929b	*E. coli*	*bla*_CTX−M−1_	Plasmid	I1α	pST35	IS*Ecp1*
13	C-29945	*E. coli*	*bla*_SHV−12_	Plasmid	I1α	pST227	IS*26*
14	C-31162	*E. coli*	*bla*_CTX−M−24_	Plasmid	N	pST3	IS*Ecp1*
15	C-42978	*E. coli*	*bla*_CMY−2_	Plasmid	I1α	pST2/pCC2	IS*Ecp1*
16	C-43494	*E. coli*	*bla*_CMY−2_	Chromosome	–	NA	IS*Ecp1*
17	C-44647	*E. cloacae*	*bla*_CTX−M−15_	Chromosome	–	NA	IS*Ecp1*
18	C-45577	*E. coli*	*bla*_CTX−M−1_	Plasmid	N	ST1	IS*Ecp1*
19	C-50954	*E. coli*	*bla*_CTX−M−14b_	Chromosome	–	–	IS*Ecp1*
20	C-51026	*E. coli*	*bla*_CTX−M−1_	Plasmid	K	NA	IS*Ecp1*
21^§^	P-12356a	*E. coli*	*bla*_CTX−M−1_	Plasmid	I1α	pST3/pCC3	IS*Ecp1*
	P-12356b	*E. coli*	*bla*_CTX−M−1_	Plasmid	I1α	pST7/pCC7	IS*Ecp1*
22	P-13127	*E. coli*	*bla*_CTX−M−1_	Plasmid	I1α	pST58/pCC58	IS*Ecp1*
23	P-13277	*E. coli*	*bla*_CTX−M−27_	Plasmid	F	F2:A-:B-	IS*26*
24	P-14152	*E. coli*	*bla*_CTX−M−14var_	Plasmid	F	F24:A-:B1	IS*Ecp1*
25	P-14808	*E. coli*	*bla*_CTX−M−1_	Plasmid	I1α	pST3/pCC3	IS*Ecp1*
26	P-15052a	*E. coli*	*bla*_CTX−M−15_	Plasmid	F	F2:A4:B1	IS*Ecp1*
27	P-15274	*E. coli*	*bla*_CTX−M−1_	Plasmid	I1α	pST3/pCC3	IS*Ecp1*
28	P-16235	*E. coli*	*bla*_CTX−M−1_	Plasmid	X1	NA	IS*26*
29	P-16817	*E. coli*	*bla*_CTX−M−15_	Plasmid	F	F1:A1:B1	IS*26*
30	P-18176	*E. coli*	*bla*_CTX−M−15_	Chromosome	–	–	IS*26*
31	P-18216	*E. coli*	*bla*_CTX−M−2_	Plasmid	HI1	NA	IS*CR1*
32	P-19659	*E. coli*	*bla*_SHV−12_	Plasmid	I1α	pST3/pCC3	IS*26*
33	P-20005	*E. coli*	*bla*_CTX−M−15_	Plasmid	F	F1:A4:B1	IS*Ecp1*
34	P-20371	*E. coli*	*bla*_CTX−M−15_	Plasmid	I1α	pST188	IS*Ecp1*
35	P-21458	*E. coli*	*bla*_CTX−M−1_	Plasmid	I1α	pST58/pCC58	IS*Ecp1*
36	P-23698	*E. coli*	*bla*_SHV−12_	Plasmid	N	pST1	IS*26*
37	P-23883	*E. coli*	*bla*_CTX−M−15_	Chromosome	–	–	IS*Ecp1*
38	P-25030	*E. coli*	*bla*_DHA−1_	Plasmid	NT		NP
39	P-26355	*E. coli*	*bla*_CTX−M−14_	Plasmid	F	F2:A-:B-	NP
40	P-26492	*E. coli*	*bla*_CMY−2_	Plasmid	I1α	pST12/pCC12	IS*Ecp1*
41	P-26517	*E. coli*	*bla*_CTX−M−14_	Plasmid	B/O	NA	IS*Ecp1*
42	P-28847	*E. coli*	*bla*_CTX−M−27_	Plasmid	F-R	F1:A2:B20	IS*26*
43^§^	P-29344a	*E. coli*	*bla*_CTX−M−15_	Plasmid	F	F4:A-:B-	IS*Ecp1*
	P-29344b	*E. coli*	*bla*_CTX−M−15_	Plasmid	F	F2:A1:B1	IS*Ecp1*
44	P-29754	*E. coli*	*bla*_CTX−M−1_	Plasmid	I1α	pST3/pCC3	IS*Ecp1*
45	P-30462	*E. coli*	*bla*_CTX−M−15_	Plasmid	I1α	pST31/pCC31	IS*Ecp1*
46	P-30656	*E. coli*	*bla*_CTX−M−14_	Plasmid	B/O	NA	IS*Ecp1*
47	P-41705	*K. pneumoniae*	*bla*_CTX−M−15_	Plasmid	HIB-M	NA	IS*Ecp1*
48	P-44471	*E. coli*	*bla*_CTX−M−15_	Chromosome	–	–	IS*Ecp1*
49	P-45037	*E. coli*	*bla*_CTX−M−15_	Plasmid	I1α	pST1	IS*Ecp1*
50	P-45865	*E. coli*	*bla*_CTX−M−15_	Plasmid	K	NA	IS*Ecp1*
51	P-45995	*E. coli*	*bla*_CTX−M−15_	Plasmid	F	F2:A4:B1	IS*Ecp1*
52	P-50908	*E. coli*	*bla*_CMY−2_	Plasmid	K	NA	IS*Ecp1*

*C, child; P, parent; ST/CC, sequence type/clonal complex; pST/pCC, plasmid sequence type/plasmid clonal complex; ND, not determined; NA, not available; NP, not present; NT, non typeable plasmid*.

* *Letters a and b indicate isolates with distinct colony morphotypes originated from the same fecal sample*.

∧ *Gene typing derived from (van den Bunt et al., 2016)*.

§ *ST/CC was determined for E. coli isolates recovered from the same fecal sample as distinctive colonial morphotypes: C-29929a and b (345 and 131/131, respectively), P-12356a and b (1775 and 648/648, respectively), and P-29344a and b (10/10, both)*.

**Table 2:** Molecular characteristics of the ESC^R^ *Enterobacteriaceae* recovered from child-parent pairs in 14 Dutch households.

**Household**	Isolate^*^	**Bacterial species**	**ST/CC**	**ESBL/AmpC gene^∧^**	**Location**	**Plasmid rep/inc-type**	**Plasmid subtype**	**Insertion sequence**
53	C1a	*E. coli*	1380	*bla*_CTX−M−15_	Plasmid	K	NA	NP
	C1b	*E. coli*	34/10	*bla*_CTX−M−15_	Plasmid	F	F1:A1:B16	IS*Ecp1*
	P1	*E. coli*	3036	*bla*_CTX−M−15_; *bla*_DHA−1_	Plasmid	I1α^#^	pST68/pCC31	IS*26*
54	C2, P2	*E. coli*	131/131	*bla*_TEM−52c_	Plasmid	I1α	pST36/pCC5	NP
55	C3a	*E. coli*	38/38	*bla*_CTX−M−14b_	Plasmid	F	F29:A4:B10	IS*Ecp1*
	C3b	*E. coli*	93/168	*bla*_CTX−M−15_	Plasmid	F	ND	IS*Ecp1*
	P3a	*E. coli*	10/10	*bla*_CTX−M−3_	Plasmid	I1α	pST57	IS*Ecp1*
	P3b	*E. coli*	3610	*bla*_CTX−M−14b_	Chromosome	-	-	IS*Ecp1*
56	C4, P4	*E. coli*	301/165	*bla*_CTX−M−14_	Plasmid	K	NA	IS*Ecp1*
57	C5	*E. coli*	131/131	*bla*_CTX−M−15_	Chromosome	-	-	IS*Ecp1*
	P5	*K. pneumoniae*	570	*bla*_CTX−M−15_	Plasmid	F	ND	IS*Ecp1*
58	C6	*E. coli*	131/131	*bla*_CTX−M−3_	Plasmid	F	F2:A-:B-	IS*Ecp1*
	P6	*E. coli*	131/131	*bla*_CTX−M−3_	Plasmid	F	F29:A-:B-	IS*Ecp1*
59	C7	*K. pneumoniae*	48	*bla*_CTX−M−15_	Chromosome	-	-	IS*Ecp1*
	P7	*E. coli*	69/69	*bla*_CTX−M−15_	Chromosome	-	-	IS*Ecp1*
60	C8	*E. coli*	1312	*bla*_CTX−M−15_	Plasmid	colE	NA	IS*Ecp1*
	P8	*E. coli*	23	*bla*_CTX−M−1_	Plasmid	I1α	pST190	IS*Ecp1*
61	C9, P9	*E. coli*	131/131	*bla*_CTX−M−3_	Plasmid	Y	NA	IS*Ecp1*
62	C10, P10a, P10b	*E. coli*	38/38	*bla*_CTX−M−14_	Chromosome	-	-	IS*Ecp1*
63	C11, P11	*E. coli*	665	*bla*_SHV−12_	Plasmid	I1α	pST95	IS*26*
64	C12	*E. coli*	10/10	*bla*_SHV−12_	Plasmid	I1α	pST228	IS*26*
	P12	*E. coli*	69/69	*bla*_CTX−M−27_	Plasmid	F	F95:A-:B1	IS*26*
65	C13, P13	*E. coli*	69/69	*bla*_CTX−M−14_	Plasmid	B/O	NA	IS*Ecp1*
66	C14, P14	*E. coli*	69/69	*bla*_CTX−M−27_	Plasmid	F	F1:A2:B20	IS*26*

*C, child; P, parent; ST/CC, sequence type/clonal complex; pST/pCC, plasmid sequence type/plasmid clonal complex; ND, not determined; NA, not available; NP, not present*.

* *Letters a and b indicate isolates with distinct colony morphotypes originated from the same fecal sample*.

∧ *Gene typing performed in van den Bunt et al. (2016)*.

# *IncI1α carries bla_CTX−M15_ only. Transformants carrying bla_DHA−1_ were not recovered (performed in duplicate)*.

### Plasmid typing and insertion sequence analysis

Total bacterial DNA was extracted using the DNeasy Blood and Tissue kit (QIAGEN, Hilden, Germany) according to manufacturer's recommendations. Plasmid DNA was extracted using the alkaline lysis method and transformed into DH10B cells via electroporation (Invitrogen, Van Allen Way, CA USA) (Liakopoulos et al., 2016).

Transformants were selected on LB agar plates supplemented with 1 mg/L cefotaxime and tested for the presence of the ESBL/AmpC gene of the corresponding donor isolate by PCR, as previously described (van den Bunt et al., 2016). Replicon typing of each ESBL/AmpC-encoding plasmid was determined with the PBRT KIT (DIATHEVA, Fano, Italy) according to manufacturer's recommendations, with the addition of single PCRs for IncX4 and ColE plasmids, as previously described (García-Fernández et al., 2009; Johnson et al., 2012). Subtyping of plasmids belonging to replicon types for which subtyping schemes are available (F, HI2, I1α/γ, and N), was performed as previously described (García-Fernández et al., 2008, 2011; Garcia-Fernandez and Carattoli, 2010; Villa et al., 2010). When necessary, chromosomal location of the ESBL/AmpC genes was confirmed by I-Ceu-I-PFGE of total DNA followed by Southern blot hybridization with intragenic β-lactamase and 16S rDNA and/or S1-PFGE with intragenic β-lactamase probes, as previously described (Liu et al., 1993; Barton et al., 1995).

The presence of frequent insertion sequences (IS) IS*CR1*, IS*Ecp1* and IS*26* in the immediate upstream region of the ESBL/AmpC genes was determined for all ESC^R^ isolates by PCR using combinations of primers for IS and ESBL/AmpC genes, as previously described (Liakopoulos et al., 2016).

### Genetic relatedness and clonal analysis

*E. coli* and *K. pneumoniae* isolates were characterized by multi-locus sequence typing (MLST), as previously described (Diancourt et al., 2005; Wirth et al., 2006). *E. coli* isolates belonging to the same sequence type (ST) and recovered from child and parent isolates from the same household, were assessed for genetic relatedness by PFGE of XbaI-digested genomic DNA using a CHEF DR-III apparatus (Bio-Rad Laboratories, Hercules, CA, USA) following the standardized protocol of PulseNet (Ribot et al., 2006). XbaI-digested genomic DNA from *Salmonella enterica* serotype Braenderup strain H9812 was used as a molecular reference marker (Hunter et al., 2005). Cluster analysis was performed using BioNumerics, version 6.6 (Applied Maths, Sint-Martens-Latem, Belgium) as previously described (Liakopoulos et al., 2016).

### Intra-familial co-colonization

In this study we followed a three-step approach to define intra-familial co-colonization: (i) is there any association between parent and child colonization; (ii) if yes, are they sharing the same source or just same risk factors and (iii) did the colonization with an identical strain occur by chance or are parent and child sharing the same source. Briefly, based on the observed prevalence of ESC^R^ *Enterobacteriaceae* in children and parents, and assuming colonization in children and parents was uncorrelated, we determined the probability that both parent and child in a given household were both colonized by an ESC^R^ *Enterobacteriaceae*. Family members exposed to the same source are expected to be colonized by identical ESC^R^ *Enterobacteriaceae*, defined as isolates belonging to the same ST/PFGE-pattern and carrying an identical ESBL/AmpC-gene on the same genetic location [plasmid type (and subtype) or the chromosome]. On the other hand, family members sharing risk factors are less likely to be colonized by an identical ESC^R^ *Enterobacteriaceae*. Hence, we subsequently determined the probability that both parent and child carried an identical isolate with the same ESBL/AmpC-gene on the same genetic location. All expected values were compared to the observed ones by binomial probability testing. See Data Sheet 1 in Supplementary Material for more information on the calculations.

In addition, characteristics of the households with identical ESBL/AmpC-gene type were compared to the non-identical households by Fisher's exact test. Binomial probability testing and Fisher's exact tests were performed in STATA 13 (StataCorp LP, College Station, TX, USA) and *P*-values < 0.05 were considered as statistically significant.

## Results

### Plasmid, insertion sequence, and ESBL/AmpC gene association

Results of genomic localization of ESBL/AmpC genes found in 87 ESC^R^ *Enterobacteriaceae* are summarized in Table S1. Overall, most of the genes were plasmid located, independent on source of isolation (child or parent) and bacterial species.

Among ESBL/AmpC genes from preschool children isolates, the majority (75.7%) was encoded on plasmids belonging to 10 different replicon types or non-typeable ones. Each replicon type was associated with one to four different ESBL/AmpC genes. The most predominant was IncI1α (35.7%; *n* = 10) associated with *bla*_SHV−12_ (*n* = 5), *bla*_CTX−M−1_ (*n* = 2), *bla*_CMY−2_ (*n* = 2) or *bla*_TEM−52c_ (*n* = 1), followed by IncF (21.4%; *n* = 6) associated with *bla*_CTX−M−15_ (*n* = 3), *bla*_CTX−M−3_ (*n* = 1), *bla*_CTX−M−14b_ (*n* = 1) or *bla*_CTX−M−27_ (*n* = 1). Genes *bla*_CTX−M−3_, *bla*_CTX−M−15_, *bla*_CTX−M−14_, *bla*_CTX−M−14b_, and *bla*_CMY−2_ were also located on the chromosome in 24.3% (*n* = 9) of the cases.

Similarly to preschool children isolates, the majority of the ESBL/AmpC genes (86.0%) from parent isolates were encoded on plasmids belonging to 11 different replicon types or non-typeable ones (Table S1). Plasmids assigned to replicon type IncI1α were the most prevalent (39.5%; *n* = 17) in association with *bla*_CTX−M−1_ (*n* = 8), *bla*_CTX−M−15_ (*n* = 4), *bla*_SHV−12_ (*n* = 2), *bla*_CTX−M−3_ (*n* = 1), *bla*_TEM−52c_ (*n* = 1), or *bla*_CMY−2_ (*n* = 1). The second most represented replicon type, IncF (30.2%; *n* = 13), was associated with *bla*_CTX−M−15_ (*n* = 7), *bla*_CTX−M−27_ (*n* = 3), *bla*_CTX−M−3_ (*n* = 1), *bla*_CTX−M−14_ (*n* = 1) or *bla*_CTX−M−14var_ (*n* = 1). A small proportion of genes (14.0%; *n* = 7), namely *bla*_CTX−M−15_, *bla*_CTX−M−14_, and *bla*_CTX−M−14b_, was encoded on the chromosome.

**Figure 1:**
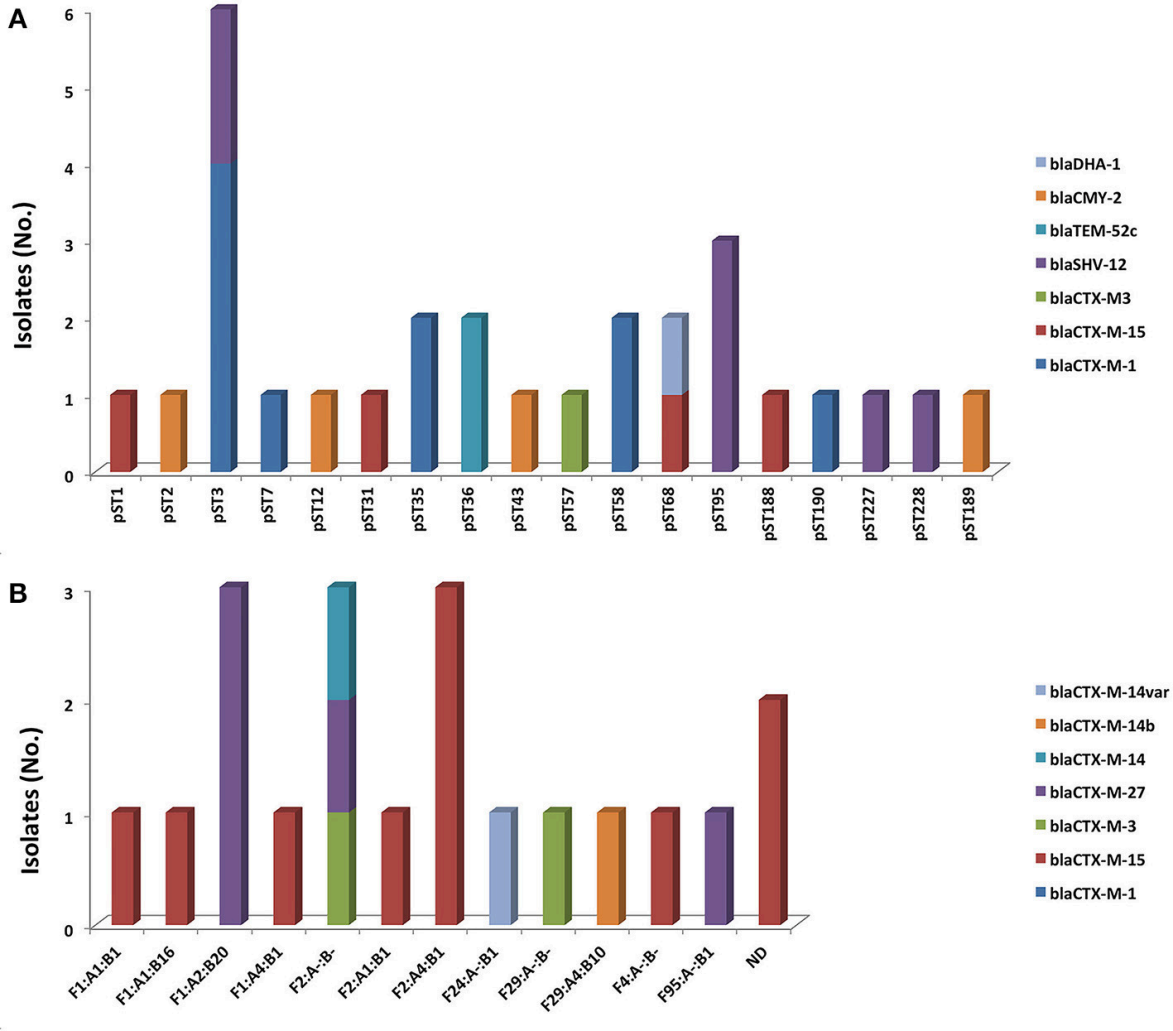
Association between ESBL/AmpC genes with **(A)** IncI1α/γ plasmid STs and **(B)** IncF replicon sequence types.

Subtyping of IncI1α/γ plasmids from both parents and children isolates revealed the presence of 28 plasmid sequence types (pST): 27 IncI1α and 1 IncI1γ types, each encoding one to two ESBL/AmpC genes. The most predominant types were pST3 (20.7%; *n* = 6) and pST9 (10.3%; *n* = 3), mostly associated with *bla*_CTX−M−1_ and *bla*_SHV−12_ (Figure 1A). Subtyping of IncF plasmids assigned them to 12 different replicon sequence types (RST) encoding one to three ESBL/AmpC genes (Figure 1B). The most prevalent ones were F1:A2:B20 (15.0%; *n* = 3), F2:A-:B- (15.0%; *n* = 3) and F2:A4:B1 (15.0%; *n* = 3) in association with *bla*_CTX−M−27_, *bla*_CTX−M−14_, *bla*_CTX−M−3_, and *bla*_CTX−M−15_.

Eighty-one (93%) of the ESBL/AmpC genes under investigation were associated with insertion elements IS*Ecp1* (*n* = 62), IS*26* (*n* = 17) or IS*CR1* (*n* = 2; Table S1). IS*Ecp1* was mostly associated with ESBL genes belonging to CTX-M-1-group in both children (*n* = 16) and parents (*n* = 25), as well as with genes of the CTX-M-9-group (*n* = 7 and *n* = 8, respectively) and *bla*_CMY−2_ (*n* = 4 and *n* = 2, respectively). IS*26* was 100% associated with *bla*_SHV−12_ both in children (*n* = 5) and parents (*n* = 3), while IS*CR1* was detected only in 2 *E. coli* isolates from parents in association with *bla*_CTX−M−2_ or *bla*_CTX−M−15_.

*E. coli* isolates recovered from the same fecal sample could either belong to the same lineage (household 43) or to different STs carrying the same IncI1α plasmid encoding *bla*_CTX−M−1_ (households 12 and 21).

Variability in ST, ESBL/AmpC genes, as well as in plasmid and IS distribution for fifty-five isolates recovered either from parent or child within given households are reported in Table 1.

### Child-parent pairs

Thirty-two ESC^R^ isolates, mostly *E. coli*, recovered from parent and child within the same household (*n* = 14), including isolates of the same species recovered from the same fecal sample as distinctive colonial morphotypes, were further characterized based on their genetic relatedness, plasmid replicon and IS type (Table 2). Based on these additional criteria, intra-familial co-colonization was recalculated.

ESC^R^ *E. coli* recovered from children and parents were found to belong to 14 partially overlapping STs (Table 2). Each of the 11 STs among children was comprised of one to five isolates and associated with one to four different ESBL/AmpC genes; among parents different STs included one to four isolates and one to three ESBL/AmpC genes. Six common STs were identified between children and parents, namely ST10, ST38, ST69, ST131, ST301, and ST665. The most predominant ST among children was ST131 (*n* = 4) associated with *bla*_CTX−M−3_ (*n* = 2), *bla*_CTX−M−15_ (*n* = 1) or *bla*_TEM−52c_ (*n* = 1). Among *E. coli* isolates derived from parents, the most predominant STs were ST131 (*n* = 3) associated with *bla*_CTX−M−3_ (*n* = 2) or *bla*_TEM−52c_ (*n* = 1), and ST69 (*n* = 4) encoding *bla*_CTX−M−27_ (*n* = 2), *bla*_CTX−M−14_ (*n* = 1) or *bla*_CTX−M−15_ (*n* = 1).

In 12 of 14 households we documented co-colonization of both child and parent by either the same ESC^R^ bacterial species or different ESC^R^ *Enterobacteriaceae* encoding the same acquired ESBL/AmpC gene (Table 2). Only 10 households were co-colonized by same bacterial species encoding the same ESBL/AmpC gene.

Participants belonging to seven households were found to be co-colonized by *E. coli* isolates of the same ST encoding identical ESBL/AmpC gene on the same genetic location, either chromosome (household 62) or plasmid belonging to the same replicon type and subtype (households 54, 56, 61, 63, 65, 66; Table 2). MLST genetic relatedness among paired child and parent isolates was confirmed by XbaI-PFGE profiles (Figure S1). These seven child-parent pairs had Dutch nationality, and the households contained either one (*n* = 2; 28.6%) or two children (*n* = 5; 71.4%). In five of the households companion animals were present, and in six households one child attended day-care, which was not necessarily the child under study. Median ages were 33 years [interquartile range (IQR) 30-35] in parents, and 25 months in children (IQR 12-36). From the seven ESBL/AmpC-positive parents, five were female, which is comparable to the overall female-male-distribution of participating parents in the study (van den Bunt et al., 2016), whereas one of the parents (14.3%) used antibiotics in the 6 months previous to sampling, compared to 3.2% of the total participating parents. From the seven children, two were female, which is lower compared to the overall female-male-distribution in children (50.2%); 5 attended day-care, in line with the percentage among total children (52.0%). None of the 7 children used antibiotics in the past 6 months, compared to 7.6 of the 983 children.

Comparison between parent-child pairs colonized by non-identical or identical ESC^R^ *Enterobacteriaceae* (Table 3), revealed that within the latter there were more households with 2 children (71.4% vs. 42.9%), more children in the household attended day-care (85.7% vs. 57.1), more often companion animals were present (71.4 versus 28.6) and more parents worked (healthcare-related) with children (28.6% vs. 0.0%). None of the differences observed between the two groups were statistically significant.

**Table 3:** Comparison in characteristics of households with identical and non-identical ESC^R^ *Enterobacteriaceae*.

Variable^*^	**Household**	
	Identical^**^ (*n* = 7) n (%)	**Non-identical (*n* = 7) n (%)**
Age of the child		
<=12 months	2 (28.6)	0 (0.0)
13-36 months	4 (57.1)	5 (71.4)
37-48 months	1 (14.3)	2 (28.6)
Age of the parent		
<=34	5 (71.4)	2 (28.6)
>34	2 (28.6)	5 (71.4)
Gender of the child (male)	5 (71.4)	1 (14.3)
Gender of the parent (male)	2 (28.6)	3 (42.9)
Dutch nationality of the household	7 (100.0)	6 (85.7)
Number of children in the household		
1 child	2 (28.6)	4 (57.1)
2 children	5 (71.4)	3 (42.9)
Participating child attending day-care	5 (71.4)	4 (57.1)
A child in the household attends day-care	6 (85.7)	4 (57.1)
Animals in the household	5 (71.4)	2 (28.6)
Child uses antimicrobials	0 (0.0)	0 (0.0)
Parent uses antimicrobials	1 (14.3)	0 (0.0)
Parent works with children (healthcare related)	2 (28.6)	0 (0.0)

* *Fisher exact test was performed to test differences between households where child and parent were colonized with identical and non-identical ESC^R^ Enterobacteriaceae. None of the differences observed were statistically significant*.

** *The term identical is used to define ESC^R^ Enterobacteriaceae assigned to the same ST/PFGE-pattern within the species carrying an identical ESBL/AmpC-gene on the same genetic location [plasmid type (and subtype) or the chromosome]*.

### Intra-familial co-colonization

Given the 983 households and the observed prevalence in children and parents, the expected prevalence of co-colonization within a household in a one-to-one relationship purely based on chance was calculated here to be 0.16%. This corresponds to an expected 1.6 out of 983 households, in which both parent and child were carrying the same ESBL/AmpC gene. Extended molecular characterization of the ESC^R^ isolates performed in this study showed that 7 households with identical STs/PFGE-patterns encoded identical ESBL/AmpC genes on the same genetic location. The observed co-colonization (*n* = 7) was significantly higher than the expected one (*n* = 1.6; *P* < 0.002).

The probability of carrying an isolate with identical STs/PFGE-patterns, ESBL/AmpC-gene type and genetic location in children and parents were 0.87, 0.046, and 0.14, respectively. To allow for correlations between the presence of same bacterial species, ESBL/AmpC genes and genetic location [plasmid types (and subtypes) or chromosome], we only used the entity with the highest diversity for further calculations, i.e., the genetic location of the ESBL/AmpC gene (plasmid or chromosome). We observed that 50% (*n* = 7) of the households (*n* = 14) shared the same ESBL/AmpC gene location, which is significantly higher (*P* < 0.001) than the expected number (0.046^*^14 = 0.65).

## Discussion

The diversity of *E. coli* STs found within Dutch preschool children and parents suggests that commensal *E. coli* act as reservoir of ESBL/AmpC genes. The most prevalent STs were the pandemic lineages of extraintestinal pathogenic *E. coli* (ExPEC) ST131 and ST69 known to cause urinary and bloodstream infections, among others (Riley, 2014). These STs have been associated with a competitive advantage over other *E. coli* STs owing to a combination of antimicrobial resistance and virulence determinants promoting their clonal expansion (Manges and Johnson, 2012; Riley, 2014).

The majority of the ESBL/AmpC genes was encoded on plasmids assigned to the narrow-host range I1α/γ and F replicon types, confirming the importance of these plasmid families in the dissemination of ESBL/AmpC genes within the Dutch human population (Reuland et al., 2013, 2015, 2016; van Hoek et al., 2015). Interestingly, replicon types with known wide range of hosts were not identified among the ESBL/AmpC-encoding plasmids, suggesting the limited potential diffusion of these plasmids to genera other than *Enterobacteriaceae* in the enteric cavity.

Association of IS*Ecp1* and IS*26* with *bla*_CTX−M_ and *bla*_SHV−12_, respectively, might be related to their involvement in the mobilization of these resistance genes from the chromosome of *Kluyvera* and *K. pneumoniae*, respectively (Ford and Avison, 2004; Cantón et al., 2012). Chromosomal integration of typically plasmid-encoded *bla*_CTX−M_ genes may be facilitated by the presence of IS*Ecp1*, in an attempt to lower the fitness cost derived from harboring an entire plasmid (Baltrus, 2013). This hypothesis is supported by recent findings of chromosomal IS*Ecp1*-mediated transposition of *bla*_CTX−M_ in *E. coli* (including ST38 and ST131, as observed here; Mahrouki et al., 2012; Hirai et al., 2013; Rodríguez et al., 2014; Hamamoto et al., 2016; Guenther et al., 2017). Whole genome sequencing of the isolates might provide further information on the chromosomal integration site and genetic context of the integrated *bla*_CTX−M_ genes.

Thanks to this refined molecular analysis we gained insight into the co-carriage of ESC^R^ *Enterobacteriaceae* between preschool children and their parents within the same household, compared to our previous study (van den Bunt et al., 2016). In this study, we documented co-carriage with identical ESC^R^ *Enterobacteriaceae* in 7 (8.4%) out of 983 households vs. 14 (14.4%) based on sole gene typing of the previous analysis. Yet, this co-carriage was more frequent than expected based on pure chance, leading to the hypothesis that clonal transmission occurred between children and parents within these households. Although the ESC^R^ *E. coli* strains recovered from these households belonged to human-related STs, we cannot rule out the exposure of child and parent pairs to a common source, as we did not investigate other sources such as food and companion animals.

The high diversity of ESBL/AmpC genes, plasmid replicon types (and subtypes) and/or STs observed in the remaining households between colonized children and parents of the same households, hints to unrelated acquisitions and same risk factors (e.g., traveling, improper hand hygiene) rather than same source (e.g., either a household member or same contaminated food source). Overall, although high prevalence of intra-familial co-colonization was observed, underestimation cannot be excluded since only one of the parents was sampled from each household.

In conclusion, we calculated that even within epidemiologically linked cases, considering only ESC^R^ isolates encoding the same ESBL/AmpC gene as an indication of co-colonization, a statistically significant overestimation of the prevalence of true co-colonization was observed. We therefore argue that a potential transmission event from a preschool child to its parent or vice versa can only be assumed if at least the ESBL/AmpC gene and the encoding plasmid replicon type (and subtype) are identical between the *Enterobacteriaceae* recovered from both parent and child.

## Author contributions

AL, YG, and MT: Data acquisition; AL, GvdB, and MB: Data analysis and interpretation; AL and DC: Manuscript preparation. All authors discussed, read, contributed to, and approved the final manuscript.

## Author's note

This work was partially presented at the 25th European Congress of Clinical Microbiology and Infectious Diseases (ECCMID), 25–28 April 2015, Copenhagen, Denmark (Poster number P0958).

### Conflict of interest statement

The authors declare that the research was conducted in the absence of any commercial or financial relationships that could be construed as a potential conflict of interest.
