# Supplementary material for: High Prevalence of Intra-Familial Co-colonization by Extended-Spectrum Cephalosporin Resistant Enterobacteriaceae in Preschool Children and Their Parents in Dutch Households
Source: Front Microbiol. 2018 Feb 21;9:293. doi: 10.3389/fmicb.2018.00293 (PMC5826366; doi:10.3389/fmicb.2018.00293)
Supplement: Supplementary file 1 [file Table1.pdf]

Table S1. Plasmid, Insertion Sequence and ESBL/AmpC genes distribution per origin (child or parent), per bacterial species in entire dataset.

NT: not typable plasmid
NP: IS not present

Count of ESBL/pAmpC genes	Genes														
	blaCMY-2	blaCTX-M1	blaCTX-M14	blaCTX-M14b	blaCTX-M14var	blaCTX-M15	blaCTX-M2	blaCTX-M24	blaCTX-M27	blaCTX-M3	blaDHA-1	blaSHV12	blaTEM-52var	blaTEM-52c	Grand Total
**Child**	**4**	**4**	**4**	**2**		**11**		**1**	**1**	**3**		**5**	**1**	**1**	**37**
E. cloacae						***1***				***1***					***2***
chromosome						1				1					2
ISEcp1						1				1					2
E. coli	***4***	***4***	***4***	***2***		***9***		***1***	***1***	***2***		***5***	***1***	***1***	***34***
chromosome	1		2	1		2									6
ISEcp1	1		2	1		2									6
ColE						1									1
ISEcp1						1									1
IncB/O			1												1
ISEcp1			1												1
IncHI2						1									1
ISEcp1						1									1
IncI1α	2	2										5		1	10
IS26												5			5
ISEcp1	2	2													4
NP														1	1
IncI1γ	1														1
ISEcp1	1														1
IncK		1	1			1									3
ISEcp1		1	1												2
NP						1									1
IncN		1						1							2
ISEcp1		1						1							2
IncX1													1		1
IS26													1		1
IncY										1					1
ISEcp1										1					1
NT						1									1
NP						1									1
IncF						3			1	1					5
IS26									1						1
ISEcp1						3				1					4
IncFIncFII				1											1
ISEcp1				1											1
K. pneumoniae						***1***									***1***
chromosome						1									1
ISEcp1						1									1
**Parent**	**2**	**9**	**7**	**1**	**1**	**17**	**1**		**4**	**3**	**1**	**3**		**1**	**50**
E. coli	***2***	***9***	***7***	***1***	***1***	***15***	***1***		***4***	***3***	***1***	***3***		***1***	***48***
chromosome			2	1		4									7
IS26						1									1
ISEcp1			2	1		3									6
IncB/O			3												3
ISEcp1			3												3
IncI1α	1	8				4				1		2		1	17
IS26												2			2
ISCR1						1									1
ISEcp1	1	8				3				1					13
NP														1	1
IncK	1		1			1									3
ISEcp1	1		1			1									3
IncN												1			1
IS26												1			1
IncX1		1													1
IS26		1													1
IncY										1					1
ISEcp1										1					1
NT											1				1
NP											1				1
IncF			1		1	6			3	1					12
IS26						1			3						4
ISEcp1					1	5				1					7
NP			1												1
IncF-R									1						1
IS26									1						1
IncHI1							1								1
ISCR1							1								1
K. pneumoniae						***2***									***2***
IncF						1									1
ISEcp1						1									1
HIB-M						1									1
ISEcp1						1									1
**Grand Total**	**6**	**13**	**11**	**3**	**1**	**28**	**1**	**1**	**5**	**6**	**1**	**8**	**1**	**2**	**87**
