# Supplementary material for: High Prevalence of Intra-Familial Co-colonization by Extended-Spectrum Cephalosporin Resistant Enterobacteriaceae in Preschool Children and Their Parents in Dutch Households
Source: Front Microbiol. 2018 Feb 21;9:293. doi: 10.3389/fmicb.2018.00293 (PMC5826366; doi:10.3389/fmicb.2018.00293)
Supplement: Supplementary file 2 [file DataSheet1.pdf]

Supplementary material to: High prevalence of intra-familial co-colonization by extended-spectrum cephalosporin resistant Enterobacteriaceae in preschool children and their parents in Dutch households.

Liakopoulos et al.

November 13, 2017

1004 children and 995 parents participated in the study [1], of which 983 were pairs of a parent and a child, i.e., there were 21 children without a participating parent and 12 parents without a participating child. In total 46 parents carried ESC^R Enterobacteriaceae and 34 children. In 14 families, both the child and the parent carried ESC^R Enterobacteriaceae. To observe whether children and parents are more likely to be simultaneously colonized, we calculated how likely co-colonization occurs in at least 14 families given the observed prevalences of 46/995=4.6% in parents and 34/1004=3.4% in children and given that there is no correlation between colonization of a child an colonization of the parent. With these assumptions, the probability that both family members are positive is $p = 0.046 \times 0.034 = 0.16\%$. The probability to observe 14 of more co-colonized families equals $\sum_{i=14}^{n} = \binom{n}{i} p^i (1-p)^{n-i} = 1.1 \times 10^{-9}$. This strongly suggests that colonization in parents and children is correlated. This does not imply intra-family transmission or exposure to the same source as both family members may have similar risk factors for colonization.

To determine whether family members only have similar risk factors or if they are exposed to the same source, we determined how likely the family members had similar strains, similar plasmids and similar resistance genes. If we assume that there is no correlation between parents and children in the carried strain(s) and we know the frequencies of occurrence of strains in children and parents, we can calculate how likely a child and his/her parent carry, by chance, at least one similar strain given that they are both colonized. As *E. coli* was by far the most common strain, this probability was 0.87. Subsequently we repeated this analysis to determine how likely both family members carried a similar plasmid and how likely they both carried a similar gene. These probabilities were 0.046 and 0.14 respectively.

In 7 out of 14 households with co-carriage in the parent and the child, both family members shared the same bacterial species, plasmid and resistance gene. If we allow for the fact that there will be correlation beween the presence of certain plasmids, certain genes and

certain strains, we only use the entity with the highest diversity, i.e., the plasmids, for our further calculations.

The probability that at least 7 out of 14 households carried the same strain, plasmid and gene is therefore less than:

$$\sum_{i=7}^{14} \binom{14}{i} 0.046^i (1 - 0.046)^{14-i} = 1.2 \times 10^{-6}$$

This suggests common exposure to ESC^R Enterobacteriaceae within families. Whether there is intra-family transmission or exposure to a common source cannot be determined based on these data.

References

1. G van den Bunt, A Liakopoulos, DJ Mevius, Y Geurts, AC Fluit, MJM Bonten, L Mughini-Gras, and W van Pelt. Esbl/ampc-producing enterobacteriaceae in households with children of preschool age: prevalence, risk factors and co-carriage. *J Antimicrob Chemother.*, 72:589–595, 2017.
