# Supplementary material for: High Prevalence of Intra-Familial Co-colonization by Extended-Spectrum Cephalosporin Resistant Enterobacteriaceae in Preschool Children and Their Parents in Dutch Households
Source: Front Microbiol. 2018 Feb 21;9:293. doi: 10.3389/fmicb.2018.00293 (PMC5826366; doi:10.3389/fmicb.2018.00293)

Figure S1. *Xba*I–pulsed-field gel electrophoresis (PFGE) profiles of strains.
M: marker; C: child: P: parent. Numbers correspond to households in Table 2.

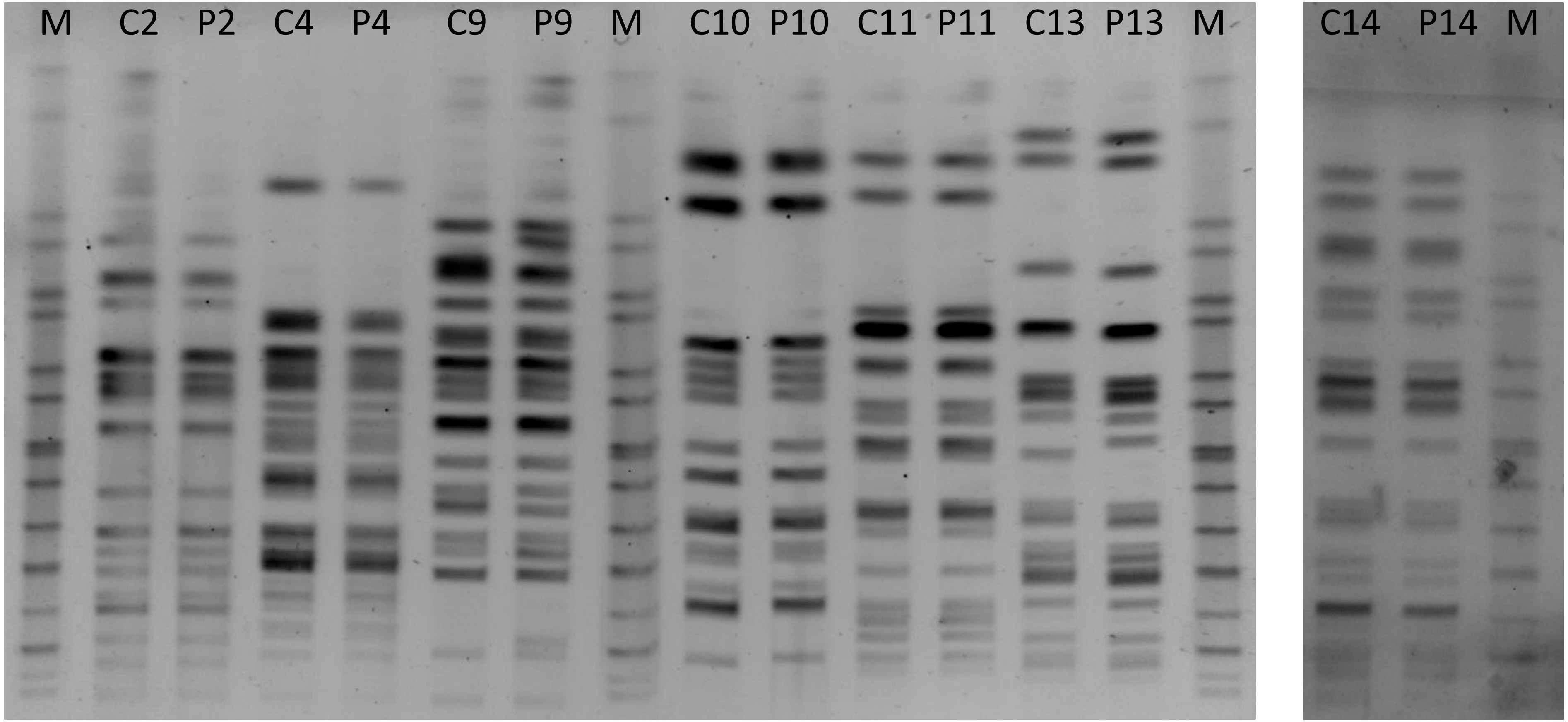

Supplement: Supplementary file 3 [file Image1.pdf]
